# Microbial community succession in the intestine of mice with deep partial-thickness burns

**DOI:** 10.3389/fmicb.2023.1140440

**Published:** 2023-04-25

**Authors:** Li-Jian Chen, Yi Liu, Jing-Wen Yang, Yan Lin, Clare Hsu, Kai-Kai Zhang, Jia-Li Liu, Jia-Hao Li, Xiu-Wen Li, Jian-Zheng Yang, Long Chen, Jia-Hao Zeng, Xiao-Li Xie, Jing-Tao Xu, Qi Wang

**Affiliations:** ^1^Guangzhou Key Laboratory of Forensic Multi-Omics for Precision Identification, School of Forensic Medicine, Southern Medical University, Guangzhou, China; ^2^Guangdong Provincial Key Laboratory of Tropical Disease Research, Department of Toxicology, School of Public Health, Southern Medical University, Guangzhou, Guangdong, China

**Keywords:** deep partial-thickness burn, 16S rRNA sequencing, microbial dysbiosis, microbial community succession, microbial diversity

## Abstract

**Introduction:**

Burn injury has been shown to lead to changes in the composition of the gut microbiome and cause other damage in patients. However, little is known about how the gut microbial community evolves in individuals who have recovered from burn injury.

**Methods:**

In this study, we established a model of deep partial-thickness burn in mice and collected fecal samples at eight time points (pre-burn, 1, 3, 5, 7, 14, 21, and 28 days post-burn) for 16S rRNA amplification and high-throughput sequencing.

**Results:**

The results of the sequencing were analyzed using measures of alpha diversity, and beta diversity and taxonomy. We observed that the richness of the gut microbiome declined from day 7 post-burn and that the principal component and microbial community structure varied over time. On day 28 after the burn, the microbiome composition largely returned to the pre-burn level, although day 5 was a turning point for change. Some probiotics, such as the Lachnospiraceae_NK4A136_group, decreased in composition after the burn but were restored in the later recovery period. In contrast, Proteobacteria showed an opposite trend, which is known to include potential pathogenic bacteria.

**Conclusion:**

These findings demonstrate gut microbial dysbiosis after burn injury and provide new insights into the burn-related dysbiosis of the gut microbiome and strategies for improving the treatment of burn injury from the perspective of the microbiota.

## 1. Introduction

Burn injuries are a common occurrence in clinical practice and have been identified as a growing threat to personal safety (Patel et al., 2018; Toppi et al., 2019). In addition to the physical damage that they can inflict, burns have also been shown to result in psychological sequelae for patients (Farag et al., 2018; Al-Ghabeesh, 2022). Despite their widespread occurrence, there is still much to be understood about the effects of burns on the human body and potential approaches for improving treatment (Moreira et al., 2018; Surowiecka et al., 2022). One area of particular interest is the impact of burns on the gut microbiome, as variations in the composition of the microbial community in the intestine have been associated with a range of health outcomes (Biagi et al., 2016; Adak and Khan, 2019).

The gut microbiome has been shown to play a role in human health and disease. It contains both beneficial and potentially harmful bacteria, which from a complex ecosystem that is important for overall health (Round and Mazmanian, 2009; Sommer et al., 2017). Studies have demonstrated differences in the gut microbial composition between healthy individuals and those with various illnesses (Rinninella et al., 2019). There have been several studies investigating the relations between the gut microbiome and burn injury. A study comparing the gastrointestinal microbiome of healthy individuals to burn patients found that burn injury is associated with microbial community imbalance (Shimizu et al., 2015). Specifically, the genera Proteus, Enterococcus and Methanobrevibacter were enriched in patients receiving enteral nutrition (Lima et al., 2021). Another study using burn injury rat models found that gut microbiota dysbiosis was reflected in changes in species richness, but not in the total number of microorganisms (Huang et al., 2017). Burn injury has also been shown to lead to microbial translocation (Earley et al., 2015), which may be related to overgrowth of gram-negative aerobic bacteria. Additionally, butyrate, which is produced mainly by gram-positive bacteria and serves as a preferred energy source for colonic epithelial cells, has been shown to be affected by burn injury (Barcenilla et al., 2000; Scheppach and Weiler, 2004).

Research on wound therapy is a well-established field, but the changes that occur in the body following burn injury, including modifications to the gut microbiota, are not fully understood. Burns are particularly susceptible to infections, as well as damage to the intestinal barrier (Earley et al., 2015). Imbalance in the gut microbiome and loss of protective function can lead to illness. The gut microbiota communicates with the host to protect the body and prevent infection (Gill et al., 2006), and there are serial changes in the gut bacteria of burned patients (Shimizu et al., 2015). However, the dynamic changes in the gut microbiota during the natural healing process following burn injury are not well understood.

**Figure 1:**
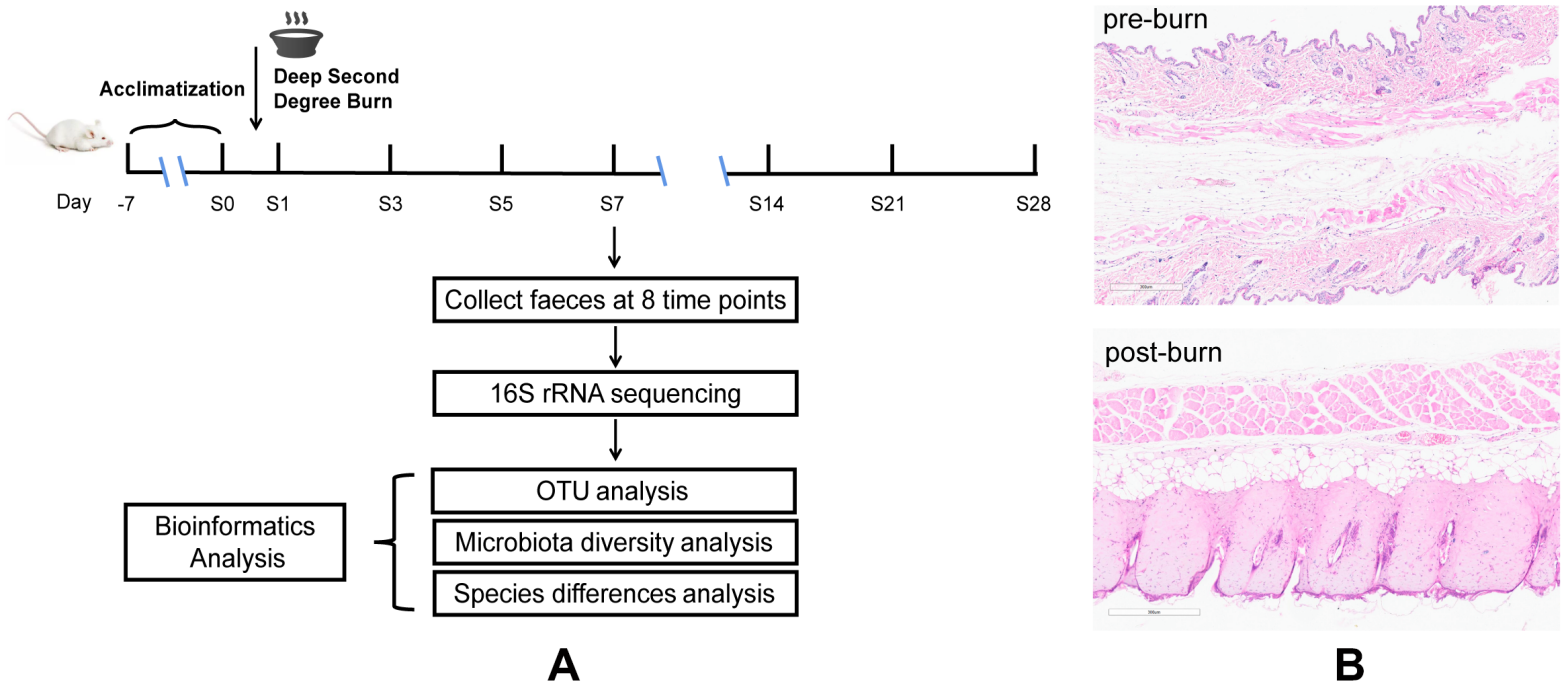
Diagrammatic sketch of the experimental protocol **(A)**, H&E for skin tissues before and after burn **(B)**.

In this study, we established a model of deep partial-thickness burn in mice and collected fecal samples for high-throughput 16S rRNA sequencing (Figure 1A). Bioinformatic analysis was performed to compare the gut microbial construction at eight time points. We observed a distinct succession in the gut microbiota of burn-injured mice over time, providing new insights into potential strategies for improving burn injury treatment from the perspective of the gut microbiome.

## 2. Materials and methods

### 2.1. Animal

Male BALB/c mice (18–20 g, 8–10 weeks old) were purchased from the Experimental Animal Center of Southern Medical University (Guangzhou, China). The experiment was approved by the Ethics Committee of Southern Medical University (Ethical Committee Approval Code:2020002). All mice were fed with standard laboratory food and water and were reared under a 12-h light/dark cycle.

### 2.2. Deep partial-thickness burn

Before the experiment, 48 mice were raised and acclimatized for 1 week. These mice were then equally divided into 8 groups at random (pre-burn, 1, 3, 5, 7, 14, 21, and 28 days post-burn, respectively, *n* = 6). Later, a model of deep partial-thickness burn injury in mice (Figure 1A) was established. Before the experiment, all mice were fasted for 12 h but provided with free access to water. Using 1% pentobarbital to anesthetize the mice half an hour before burning and then the dorsal skin of the mice was depilated and shaved (Chen Y. et al., 2021). The naked skin of mice was topically exposed (approximately 15% of body surface area) to a polyvinyl chloride (PVC) plate to scald in boiling water for 6 s (Ren et al., 2012). Following experimentation, 1 ml of saline was intraperitoneally injected into the animals initially, and then the wounds were treated with iodine and covered with Vaseline oil gauze. To prevent wound infection, the gauze dressing was changed, and the wounds were disinfected once daily. We set 8 time points, namely pre-burn and 1, 3, 5, 7, 14, 21, and 28 days post-burn. Fecal samples were collected at the indicated time points twice a day and immediately stored at −80°C before analysis. During this period, skin tissue around the wound were removed for pathological examination to confirm that the deep partial-thickness burn mouse model was established successfully. All animals were subsequently sacrificed by decapitation.

### 2.3. Histopathological observation

Dorsal skin tissue samples from the control group (1 day post-burn) and naked skin tissue exposed to boiling water from the burn group were fixed in formalin and then embedded in paraffin. Slices with 3 μm thickness were made and stained with hematoxylin and eosin (H&E) for histopathological examination (Xie et al., 2018; Ibrahim et al., 2022).

### 2.4. DNA purification, PCR amplification, and sequencing

At the start, five frozen fecal samples were randomly selected from each group for high-throughput 16S rRNA sequencing analysis. The samples were then crushed, and 100 mg of the feces was used for DNA extraction (Chen L. et al., 2021; Wang et al., 2022). Microbial DNA was extracted from the fecal samples using the E.Z.N.A.® soil DNA kit (Omega Bio-Tek, Norcross, GA, United States) according to the manufacturer’s protocols. A NanoDrop 2000 UV–vis spectrophotometer (Thermo Scientific, Wilmington, United States) was used to determine the concentration and purification of final DNA, and DNA quality was assessed using 1% agarose gel electrophoresis. Thermocycler PCR system (GeneAmp 9,700, ABI, United States) was used to amplify the bacterial 16S rRNA gene region V3-V4 with specific primers 338F (5′-ACTCCTACGG GAGGCAGCAG-3′) and 806R (5′-GGACTACHVGGGTW TCTAAT-3′). The programs of PCR reaction were as followed: 3 min of denaturation at 95°C, 27 cycles of 30 s at 95°C, 30 s for annealing at 55°C, 45 s for elongation at 72°C, and a final extension at 72°C for 10 min. PCR reactions were performed in a final value of 20 μl mixture consisting of 4 μl of 5 × FastPfu Buffer, 2 μl of 2.5 mM dNTPs, 0.8 μl of each primer (5 μM), 0.4 μl of FastPfu Polymerase and 10 ng of template DNA.

The PCR products were recovered by using a 2% agarose gel and further purified by AxyPrep DNA Gel Extraction Kit (Axygen Biosciences, Union City, CA, United States). After being eluted with Tris–Hcl, the PCR products were run and detected on a 2% agarose gel, which were then quantified using QuantiFluor ™ -ST (Promega, United States) according to the manufacturer’s protocol. Based on the standard protocols by Majorbio Bio-Pharm Technology Co. Ltd. (Shanghai, China), and the purified fragments were used to construct paired-end sequence libraries on the Illumina MiSeq platform (Illumina, San Diego, United States).

### 2.5. Bioinformatics analysis

#### 2.5.1. Operational taxonomic units analysis

The sequences were assigned to the same operational taxonomic units (OTU) with a 97% similarity using Uparse (version 7.0.1090^1^). Chomeras were detected and rejected using UCHIME to obtain the representative sequence of OTUs. The RDP classifier Bayesian algorithm was used to carry out the taxonomic classification of these OTU by using the Silva align database.^2^ In addition, the resulting table of OTUs was submitted to BugBase^3^ to predict the phenotypes of microbes. The gene functions of the sequences were systematically analyzed by Kyoto Encyclopedia of Genes and Genomes (KEGG^4^).

#### 2.5.2. Microbiota diversity analysis

The differences in the resulting sequences were characterized by α-diversity and β-diversity. The Sobs index and ACE index were used to evaluate community richness via different formulas, and the Shannon index, and Simpson index were used to evaluate community diversity. All the indices were calculated at the OTU level. The Kruskal–Wallis test was used to test for differences, without assuming that the data followed a normal distribution. Principle coordination analysis (PCoA) and principal component analysis (PCA) were used to assess β-diversity, which reflected the distance between different groups of samples based on the unweighted_unifrac or bray_curtis. Microbiota typing analysis was also applied to study the community construction of predominant microbiota within the sample.

#### 2.5.3. Species differences analysis

The communities or species contributing to sample partitioning were determined by using the linear discriminant analysis (LDA) effect size (LEfSe) method.^5^ LEfSe analysis was performed according to the following procedures: first, significantly different abundances of features between groups were detected using the Kruskal–Wallis (KW) sum-rank test; second, the different features of the previous step were tested by the Wilcoxon rank-sum test between subgroups; and last, LDA was calculated.

### 2.6. Statistical analysis

All of the differences in mean values among the 8 groups were determined by one-way analysis of variance (one-way ANOVA) using GraphPad Prism (Version 8.0.2; GraphPad Prism Software). These differences were considered statistically significant when *p* < 0.05.

## 3. Results

### 3.1. Successful establishment of deep partial-thickness burn models

Deep partial-thickness burns are characterized by damage to the entire epidermis and dermal skin structures (Bader et al., 2012). Therefore, we used HE staining to assess the success of our burn models (Figure 1B). The results showed that in the control group, the skin was preserved with orderly arranged collagen fibers. However, in the burn group (1 day post-burn), the skin displayed full-thickness necrosis in the epidermal and dermal layers, as well as disorganized collagen fibers and vacuolar formations. Additionally, we observed vasodilatation in the burn group. These findings suggest that our burn models were successfully established and mimicked the characteristics of deep partial-thickness burns.

### 3.2. 16S rRNA sequencing statistics

A total of 1,919,846 sequences and 804,159,262 bases were obtained from high-throughput 16S rRNA sequencing of 40 fecal samples, with an average length of 418 base pairs (bp). The detailed information for each sample’s sequences is provided in Supplementary Table S1, indicating the reliability of the sequencing results. Additionally, 12, 42, 66, 132 and 583 sequences at the phylum, orders, family, genus and OTU levels were identified based on species annotation, which were used for further analysis.

### 3.3. Burn injury leads to time-dependent gut microbial diversity alterations

**Figure 2:**
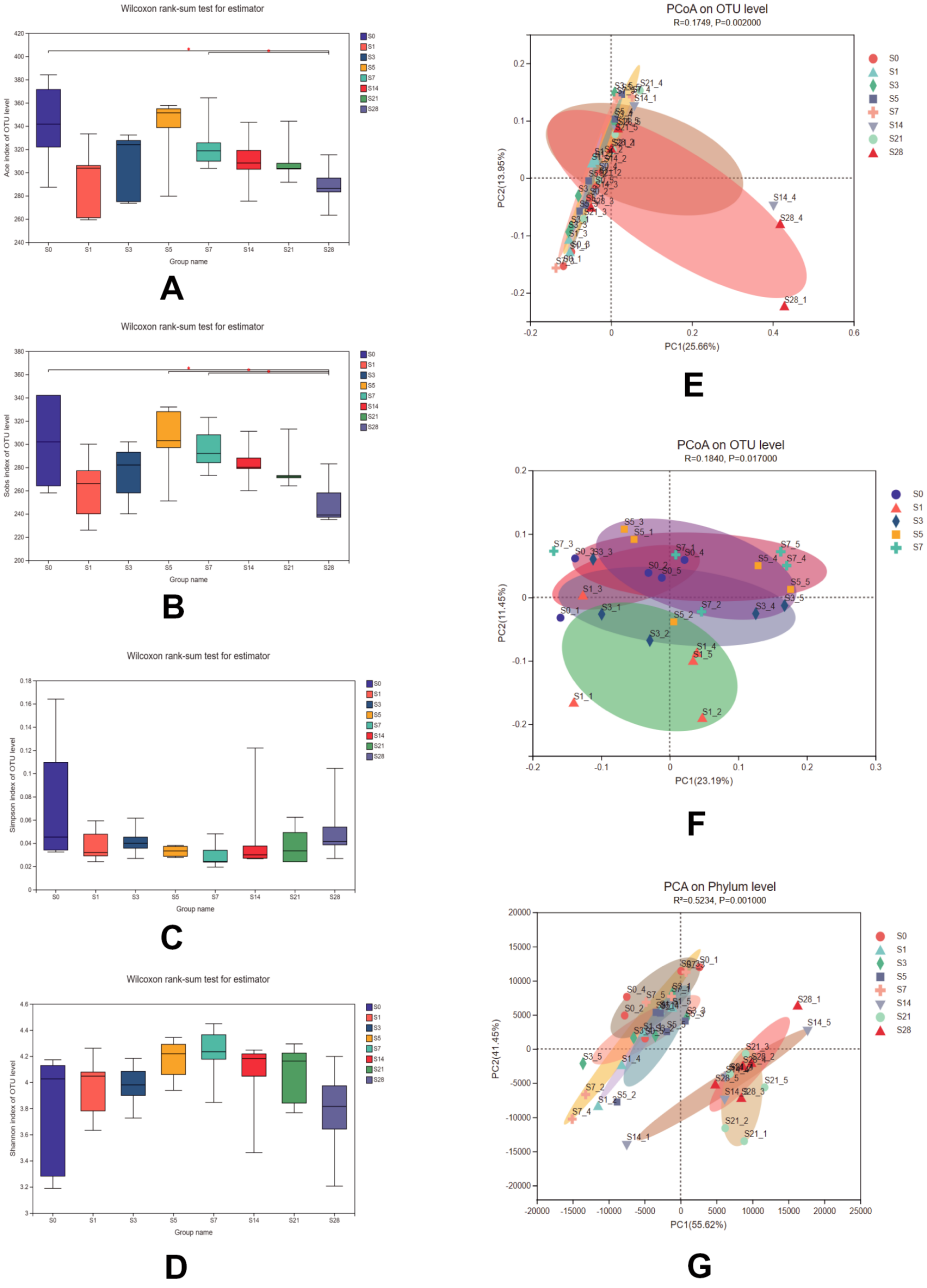
Comparison of the Ace **(A)**, Sobs **(B)**, Simpson **(C),** and Shannon **(D)** indices among the 8 groups. Data were analyzed using the Wilcoxon rank-sum test. PCoA of 8 groups **(E)** and within 1 week after burn **(F)** using unweighted_unifrac on the OTU level [ADONIS test, *R* = 0.1749, *P* = 0.002 for **(E)** and *R* = 0.1840, *P* = 0.017 for **(F)**]. **(G)** PCA of samples at the phylum level using bray_curtis (ADONIS test, *R*_2_ = 0.5234, *P* = 0.001). *n* = 5, *significantly higher (*p* < 0.05).

To assess the impact of burn injury on gut microbial diversity, we compared the α-diversity and *β*-diversity of the microbiome among the eight groups. As shown in Figures 2A–D, there were no significant differences in the Shannon and Simpson indices, but significant differences in the Ace, and Sobs indices were observed between pre-burn and post-burn samples. These two indices decreased exponentially on the first day after burn injury, and recovered gradually until day 5 post-burn and remained decreasing until day 28. These results suggest that burn injury did not alter overall diversity but rather affected the species richness of the gut microbiota, which recovered naturally over time.

Additionally, microbial communities were found to be clustered according to their respective groups, particularly on day 14 and day 28 after burn injury, as shown in the PCoA plot based on the OTU level (Figure 2E). We further analyzed the cluster within a week post-burn, and found that the discrepancies were highest on the first post-burn day, indicating an acute response to burn injury in the mice (Figure 2F). We also conducted PCA at the phylum level (Figure 2G) and found that the differences in microbial community clustering between the pre- and post-burn samples were more significant than those observed in the PCoA.

### 3.4. Burn injury leads to time-dependent gut microbial dysbiosis

**Figure 3:**
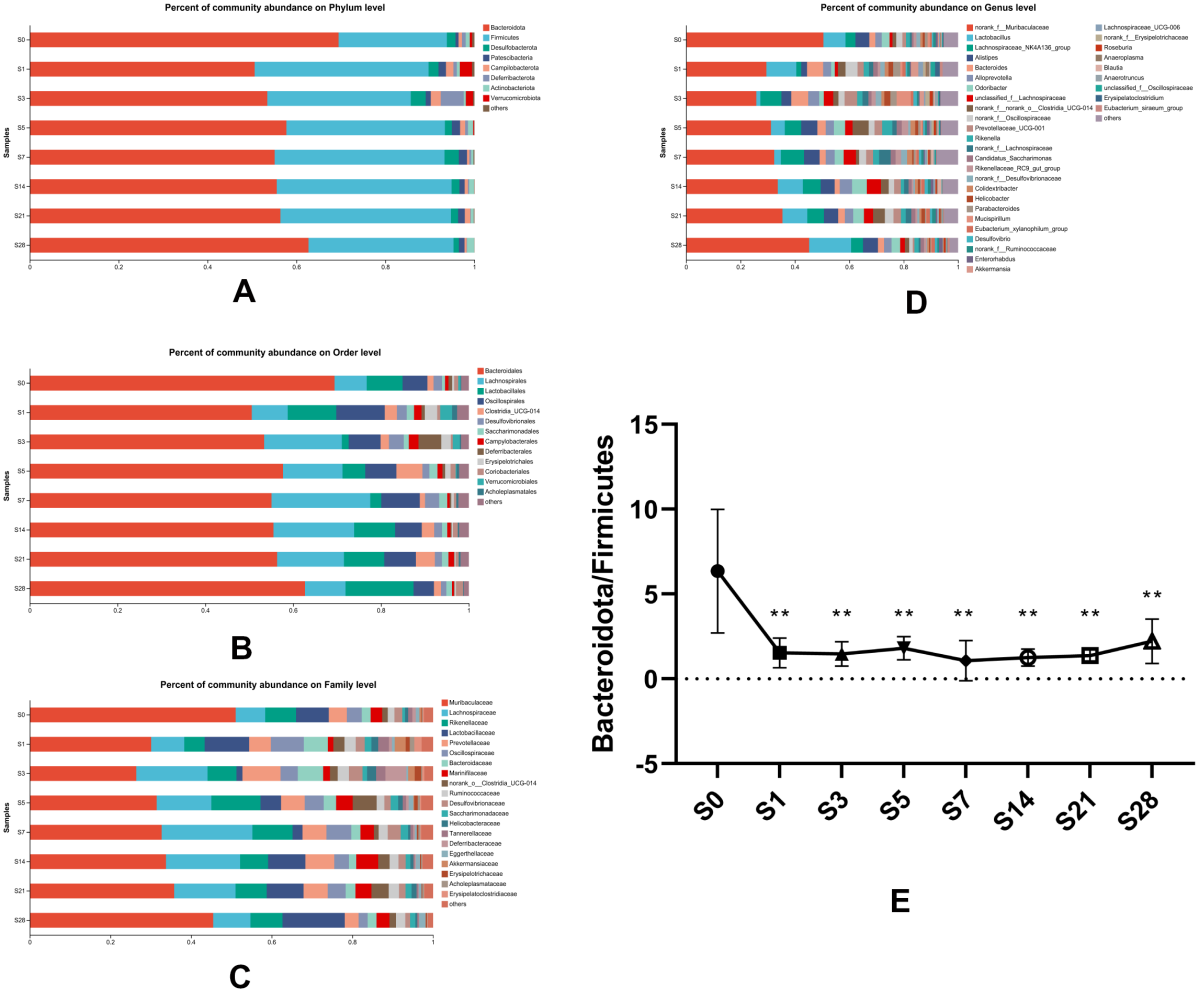
Composition of gut microbial communities altered over time. Bacterial community composition at the phylum **(A)**, order **(B)**, family **(C),** and genus **(D)** levels. Microbial abundance was calculated as a percentage of the total bacterial taxon within each sample. The bar chart shows the average value for each group. **(E)** The ratio of Bacteroidetes to Firmicutes, which was found to vary over time, showed significant differences (**p < 0.01) when each post-burn group was compared to the pre-burn group. S0, pre-burn; S1, S3, S5, S7, S14, S21, and S28 represent days 1, 3, 5, 7, 14, 21, and 28 post-burn, respectively.

To understand the changes in gut microbial community structure over time before and after burn injury, we analyzed the distribution of gut microbiota at eight time points. Our results showed that the alteration in gut microbial composition was closely correlated with burn injury at four levels of analysis (phylum level, order level, family level, germs level) (Figures 3A–D). At the phylum level, Bacteroidetes and Firmicutes were the two predominant phyla in this animal model, with Bacteroidetes comprising a slightly larger proportion than Firmicutes. As represented in Figure 3E, we analyzed the changes in the ratio of Bacteroidetes to Firmicutes and found that it decreased significantly on the first day after burn injury. This ratio fluctuated slightly over the following period and had a recovery trend to baseline by day 28 post-burn, although it did not fully recover. These findings suggest that burn injury leads to time-dependent gut microbial dysbiosis.

**Figure 4:**
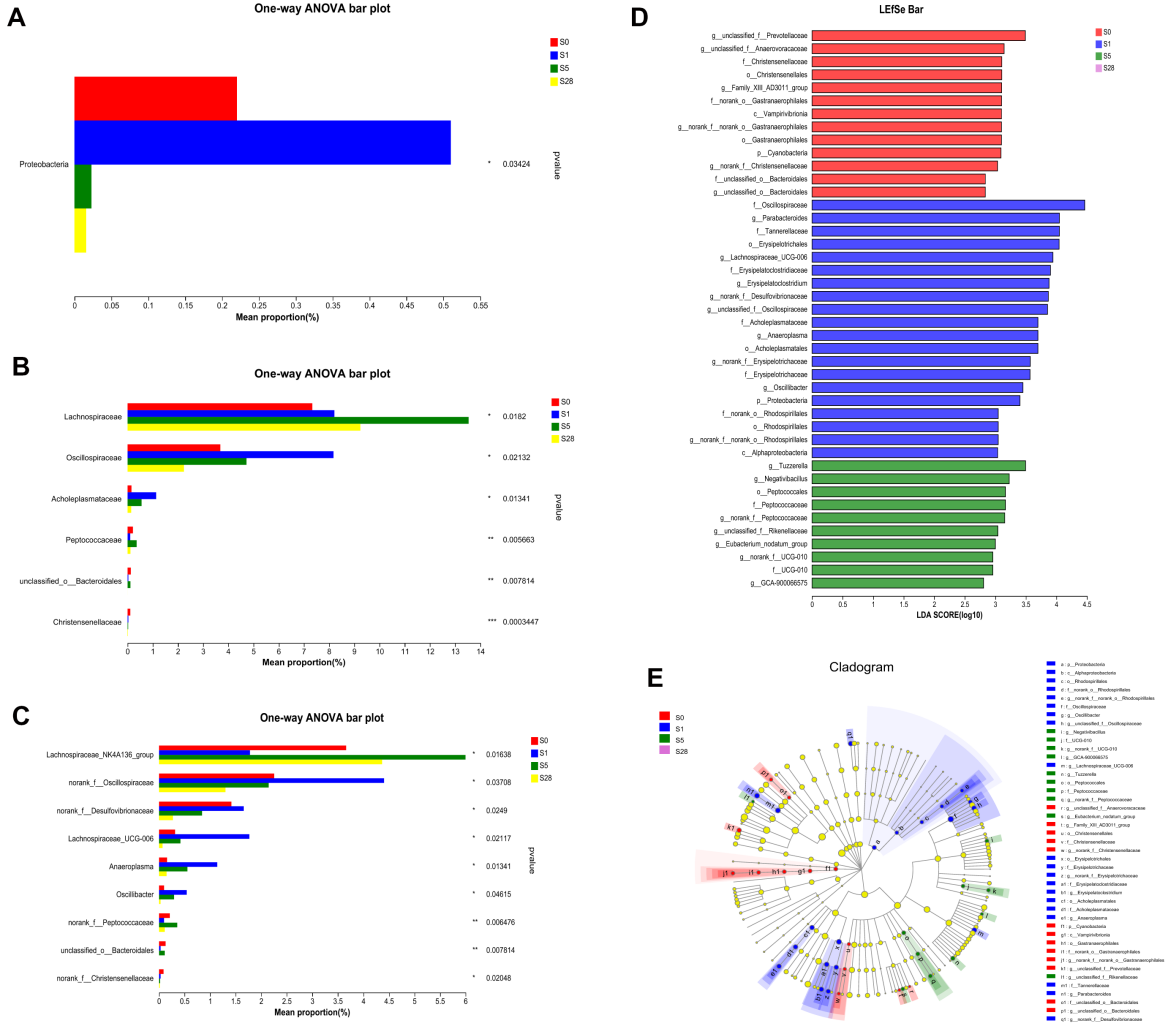
Further analysis on the day before burn injury and the days 1, 5, 28 after burn. Significance test of differential bacteria at the phylum level **(A)**, family level **(B)**, and genus level **(C)**, using one-way ANOVA, *p < 0.05, **p < 0.01, ***p < 0.001. The length of bar represents the abundance of bacteria. LDA **(D)** value distribution histogram revealed distinct taxa intestine microbiome composition. LEfSe analysis of classification level from phylum to genus, using an all-against-all (stricter) comparison strategy. Taxa with LDA values greater than 2 are presented. LEfSe cladogram **(E)** shows the different bacterial abundant taxa. Different node colors denote enrichment significance in corresponding groups, whereas the yellow color denotes no significant differences among the groups.

Our results showed that the most dramatic changes in alpha diversity and dominant phyla occurred on the first day after burn injury, suggesting that this may be a period of acute injury. The microbial alterations showed a turning point at 5 days after the burn injury, as the ratios of dominant phyla appeared to have reached a recovery status by day 5 post-burn and remained stable thereafter. These findings suggest that the composition of the gut microbial community underwent significant changes in response to burn injury. Based on these findings, we chose to focus on four time points (pre-injury, day 1, day 5, and day 28 post-injury) for further analysis of differential bacteria. Proteobacteria, which is a well-known potential pathogenic bacterium, showed a significant increase in abundance on the first day after burn injury, followed by a decrease on days 5 and 28 post-injury (Figure 4A). We observed significant increases in the abundance of Oscliospiraceae and Acholeplasmtaceae after burn injury, which partially recovered over time. In contrast, Lachnoapiraceae reached its maximum abundance on day 5 post-injury (Figure 4B). At the genus level, we observed an acute increase in the abundance of norank_f_Oscillopriaceae, norank_f_Desulfovibrionaceae, Lachnospiraceae_UCG-006, Anaeroplasma and Oscillibacter after burn injury, followed by a gradual decrease. In contrast, Lachnospiraceae_NK4A136_group showed a decrease after burn injury. Additionally, we found that the abundance of two beneficial bacteria, Christensenellaceae and unclassified_o__Bacteroidales, significantly decreased after burn injury and almost disappeared by day 28 post-burn, indicating a significant shift in the gut microbial community composition of the mice (Figure 4C).

We used LEfSe analysis to investigate changes in the microbiome from the phylum to genus levels, and the results are presented in a cladogram and bar chart (Figures 4D,E). We compared the microbial communities at four time points and found that separate clusters of the microbiota were distinguished at each time point, further supporting the conclusion that burn injury caused significant changes in microbial structure. As shown in the figure, 13, 20, and 10 bacteria made significant contributions to the separation of each group. Unclassified_f_Prevotellaceae and Christensenellales were significantly enriched before burn injury, while potential pathogens such as Lachnospiraceae _ UCG-006 and norank_f_Desulfovivrionaceae were significantly enriched on the first day after burn injury. Peptococcaceae was significantly enriched on day 5 post-burn. These findings suggest that burn injury leads to significant changes in the composition and abundance of specific microbial taxa.

### 3.5. Burn injury leads to time-dependent variation of dominant species contribution

**Figure 5:**
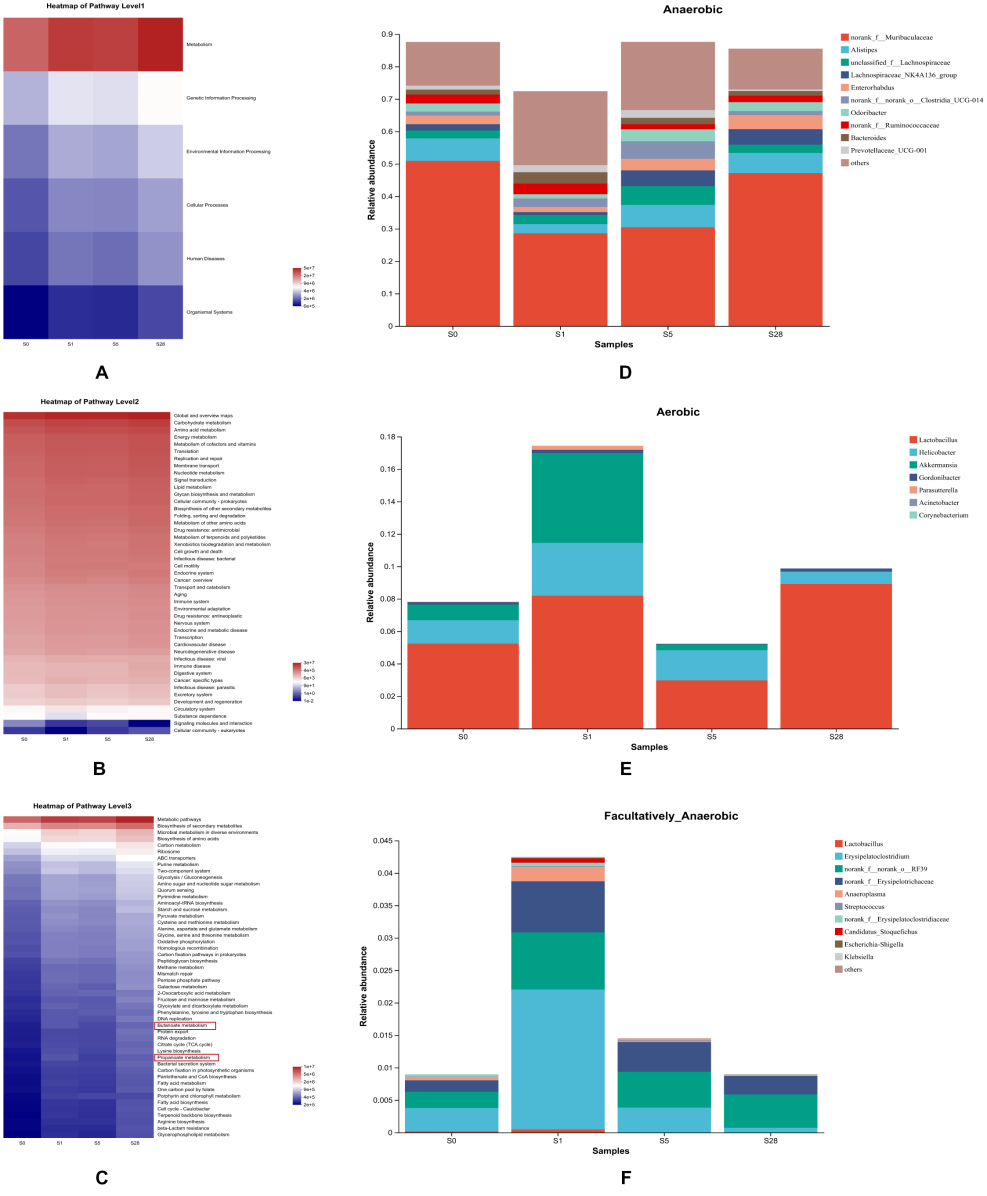
Analysis of microbial gene function prediction and dominant species contribution. Heatmap of pathways at level 1 **(A)**, level 2 **(B)**, and level 3 **(C)** determined in the KEGG pathway database. The color patch gradient is used to show the abundance change of different functions in the samples, and the legend provides a representation for the color gradient value. BugBase for anaerobic **(D)**, aerobic **(E)**, and facultatively anaerobic **(F)**, at the genus level. Each color represents a different species, and the ordinate is the contribution degree of every bacterium to this phenotype.

To further understand the impact of dominant bacterial species on the gut microbiome, we performed KEGG pathway analysis. The heatmap in Figures 5A–C shows the functional predictions based on the KEGG pathway database at four time points, and reveals time-dependent changes in pathway enrichment at three levels. We found that the post-burn period and the pre-burn time point had significant differences in enrichment. We also observed that metabolic function was significant enhanced after burn injury, particularly on day 1 and day 28 post-injury. At the third level, butanoate metabolism and propanoate metabolism were enriched. As short-chain fatty acids (SCFAs) are known to play a vital role in maintaining intestinal function, these resulted suggest that burn injury is closely related to gut microbial homeostasis in mice.

Additionally, BugBase analysis further revealed clear temporal changes in gut microbiota phenotypes. On the first day after burn injury, the proportion of aerobic and facultative anaerobes increased drastically, while that of anaerobes decreased. Over the following days, anaerobes showed a gradual increase, while the other two types showed different trends (Figures 5D–F).

## 4. Discussion

This study uncovered changes in the gut microbiota of burn-injured mice, providing strong evidence for the connection between microbiota and burn injury. By analyzing the α-diversity and β-diversity at eight time points, we found that the gut microbial structure in the late post-burn period was clearly distinct from that in the pre-burn and early post-burn periods. Cluster analysis also revealed that bacteria associated with maintain normal gut flora, such as Proteobacteria and Lachnospiraceae_NK4A136_group, had varying abundances at different time points. Furthermore, KEGG pathway prediction revealed a close relationship between the microbial community and metabolism.

In our previous research, we have identified certain markers in the skin of burn mice that can be used to identify burn responses (Liu et al., 2022; Zhang et al., 2022). Burn injury is a common trauma that often results in complications, such as wound infection, which can lead to deterioration and potentially fatal outcomes (Wang et al., 2011, 2013). Burn injury has been shown to have a direct impact on intestinal function, including immune and barrier function (Costantini et al., 2009). In addition, there was elevated level of serum IL-1β, intestinal edema and intestinal p38 MAPK activation in rats with severe burn injury (Sun et al., 2017). These findings suggest that burn injury is closely associated with the intestinal damage, which may be accompanied by the gut microbial dysbiosis (Huang et al., 2017). Therefore, in this study, we established a model of deep partial-thickness burn injury that extended into the dermis to investigate the alteration of gut microbiota and the temporal evolution of gut microbial communities.

Our findings showed that gut microbial communities remained dysregulated for 1 week after burn injury, without evident recovery, suggesting the correlation between gut microbiota and burn injury. At the phylum level, the ratio of Bacteroidetes to Firmicutes significantly changed after burn injury. As a marker of gut flora status, this ratio was reported to significantly increase in the early stage of post-burn (Huang et al., 2017). Our study results showed a decrease in the ratio of Bacteroidetes to Firmicutes in the early stage after burn injury. Proteobacteria, a phylum that includes numerous pathogenic bacteria and has the most species, was found to be the most differential bacterial community at the phylum level in our study (Gupta, 2000; Shin et al., 2015). The richness in proteobacteria increased drastically after burn injury and started to decrease significantly on day 5 post-burn, suggesting that proteobacteria was the potential component that led to intestinal injury and inflammatory response. The results showed that Lachnospiraceae_NK4A136_group and Lachnospiraceae_UCG_006 differed in abundance of different time points. Lachnospiraceae_NK4A136_group was primarily reduced on the first day, while there was a drastic increase in the Lachnospiraceae_UCG_006. This suggest that these two bacteria have a dramatic correlation with burn injury. In addition, previous research has also found alterations of these gut bacteria in mice with intestinal inflammation (Wang et al., 2019; Hu et al., 2020), indicating a close relationship between these bacteria and inflammation. These findings suggest a potential link between the observed alterations in gut microbiota and the development of inflammation in the intestinal mucosa following burn injury.

Principal component analysis revealed that the composition of gut microbiota was significant different at the early period and 2 weeks after burn injury. This may be attributed to the gradual decrease in stress response and partial recovery of the injury 2 weeks post-burn. In a previous study, burn injury was shown to induce gut dysbiosis, including alterations in the abundance of probiotic and opportunistic pathogenic organisms (Huang et al., 2017). Our study results demonstrate a clear correlation between the variation of beneficial and pathogenic bacteria and the time after burn injury. This suggests that these bacteria undergo dynamic changes in order to maintain intestinal balance during the post-burn period, indicating that the function of the gut microbiota is also time-dependent. Prediction of microbial gene function revealed that metabolism played a crucial role after burn injury. During the recovery period post-burn, metabolic pathways, particularly butanoate and propanoate metabolism, were significant enriched. SCFAs, particularly butyrate and propionate, are important links between the gut microbiome and the host and play a key role in maintaining intestinal health (Hosseini et al., 2011; Załęski et al., 2013; Louis and Flint, 2017; Zhang et al., 2021). Butyrate and propionate are mainly produced under anaerobic conditions and have a regulatory impact on energy metabolism and energy supply (He et al., 2020). Microbial phenotype analysis revealed that many anaerobes were significantly decreased due to burn injury but showed recovery in the late time after burn, potentially providing insight into the microbial succession associated with burn injury (Lovell et al., 2022).

Burn injury can be divided into four types: first-degree burn, superficial second-degree burn, deep partial-thickness burn, and third-degree burn (Guo and Yu, 2021). Deep partial-thickness burn wounds are characterized by blisters that extend into the reticular dermal layer and have a higher risk of chronic inflammation of the dermis (Lukiswanto et al., 2019), which can lead to infection or scarring. This type of burn injury was exclusively investigated in BALB/c mice in our study, and different types of burns or mouse strains may exhibit different microbiome changes, requiring further research.

## 5. Conclusion

In summary, our study demonstrated that the alteration of gut microbiota over time in deep partial-thickness burn mice results in significant decreased in microbial richness at later stages, disturbance of microbial community composition, and enrichment of metabolic pathways with altered bacteria. These findings provide novel insight into burn-related gut microbial dysbiosis form the perspective of the microbiota.

## Data availability statement

The datasets presented in this study can be found in online repositories. The names of the repository/repositories and accession number(s) can be found at: https://www.ncbi.nlm.nih.gov/, PRJNA918319.

## Ethics statement

The animal study was reviewed and approved by Ethics Committee of Southern Medical University.

## Author contributions

L-JC performed the experiments, analyzed the data, and approved the final draft. YiL analyzed the data, prepared the figures, and authored the original draft. J-WY and YaL performed the experiments. CH wrote sections of the manuscript. K-KZ, J-LL, and J-HL organized the database. X-WL, J-ZY, LC, and J-HZ performed the statistical analysis. X-LX and J-TX conceived and designed the experiments. QW conceived and designed the experiments, authored or reviewed drafts of the manuscript, and approved the final draft. All authors contributed to the article and approved the submitted version.

## Funding

This work was supported by the National Natural Science Foundation of China (grant number 81871526).

## Conflict of interest

The authors declare that the research was conducted in the absence of any commercial or financial relationships that could be construed as a potential conflict of interest.

## Publisher’s note

All claims expressed in this article are solely those of the authors and do not necessarily represent those of their affiliated organizations, or those of the publisher, the editors and the reviewers. Any product that may be evaluated in this article, or claim that may be made by its manufacturer, is not guaranteed or endorsed by the publisher.

## Footnotes

^1^ http://drive5.com/uparse/

^2^ http://www.arb-silva.de

^3^ https://bugbase.cs.umn.edu/index.html

^4^ http://www.genome.jp/kegg/

^5^ http://huttenhower.sph.harvard.edu/galaxy/root?tool_id=lefse_upload
