# Supplementary material for: Microbial community succession in the intestine of mice with deep partial-thickness burns
Source: Front Microbiol. 2023 Apr 25;14:1140440. doi: 10.3389/fmicb.2023.1140440 (PMC10167003; doi:10.3389/fmicb.2023.1140440)
Supplement: Supplementary file 1 [file Table_1.DOCX]

**Table 1.** 16S rRNA sequencing statistics for the eight group samples. S0_1 to S0_5 denotes the pre-burn samples. S1 to S28 denotes the samples of different days post-burn. Seq_num and Base_num denote the number of sequence and base. Mean_length, Min_length and Max-length denote the average, minimum and maximum sequence length of each sample.

| **Sample_name** | **Seq_num** | **Base_num** | **Mean_length** | **Min_length** | **Max_length** |
| --- | --- | --- | --- | --- | --- |
| S0_1 | 39940 | 16868454 | 422.344867 | 277 | 454 |
| S0_2 | 34804 | 14677306 | 421.713194 | 276 | 431 |
| S0_3 | 38486 | 16220935 | 421.476251 | 316 | 435 |
| S0_4 | 34564 | 14486765 | 419.128718 | 282 | 457 |
| S0_5 | 43909 | 18379760 | 418.587533 | 255 | 443 |
| S1_1 | 49823 | 20803103 | 417.540152 | 283 | 477 |
| S1_2 | 43329 | 18173372 | 419.42745 | 216 | 463 |
| S1_3 | 38666 | 16254036 | 420.370248 | 277 | 430 |
| S1_4 | 41768 | 17471401 | 418.296327 | 270 | 473 |
| S1_5 | 39794 | 16696386 | 419.570438 | 277 | 441 |
| S3_1 | 45008 | 18898326 | 419.888153 | 232 | 487 |
| S3_2 | 49051 | 20492134 | 417.771992 | 239 | 444 |
| S3_3 | 49274 | 20658522 | 419.258067 | 260 | 510 |
| S3_4 | 39381 | 16483653 | 418.568675 | 282 | 443 |
| S3_5 | 46936 | 19585113 | 417.272733 | 277 | 432 |
| S5_1 | 45437 | 19042283 | 419.091996 | 252 | 433 |
| S5_2 | 50552 | 20922934 | 413.889342 | 282 | 432 |
| S5_3 | 57394 | 24041113 | 418.878506 | 252 | 431 |
| S5_4 | 46350 | 19449936 | 419.631845 | 218 | 432 |
| S5_5 | 38522 | 16190648 | 420.296142 | 277 | 442 |
| S7_1 | 45391 | 19063420 | 419.982375 | 278 | 521 |
| S7_2 | 42299 | 17450876 | 412.560013 | 210 | 443 |
| S7_3 | 43677 | 18436674 | 422.114019 | 318 | 438 |
| S7_4 | 44177 | 18230981 | 412.680377 | 265 | 430 |
| S7_5 | 41892 | 17579699 | 419.643345 | 277 | 443 |
| S14_1 | 48620 | 20409717 | 419.780276 | 257 | 503 |
| S14_2 | 52319 | 21876288 | 418.132762 | 277 | 432 |
| S14_3 | 55874 | 23266948 | 416.418155 | 218 | 458 |
| S14_4 | 51937 | 21656072 | 416.968096 | 219 | 498 |
| S14_5 | 57277 | 24047733 | 419.84973 | 277 | 494 |
| S21_1 | 64578 | 27141338 | 420.287683 | 277 | 488 |
| S21_2 | 61708 | 25682979 | 416.201773 | 218 | 491 |
| S21_3 | 50883 | 21342038 | 419.433563 | 252 | 530 |
| S21_4 | 54904 | 22938430 | 417.7916 | 219 | 494 |
| S21_5 | 62668 | 26196823 | 418.025515 | 239 | 497 |
| S28_1 | 53399 | 22482188 | 421.022641 | 255 | 437 |
| S28_2 | 54151 | 22695123 | 419.108105 | 219 | 501 |
| S28_3 | 57958 | 24458626 | 422.006039 | 276 | 477 |
| S28_4 | 52111 | 21849503 | 419.287732 | 251 | 525 |
| S28_5 | 51035 | 21557626 | 422.408661 | 277 | 439 |
